# Self-Perceived Physical Activity and Adherence to the Mediterranean Diet in Healthy Adolescents during COVID-19: Findings from the DIMENU Pilot Study

**DOI:** 10.3390/healthcare9060622

**Published:** 2021-05-23

**Authors:** Angelo Galluccio, Giovanna Caparello, Ennio Avolio, Emanuele Manes, Simona Ferraro, Cinzia Giordano, Diego Sisci, Daniela Bonofiglio

**Affiliations:** 1 Health Center srl, 87100 Cosenza, Italy; angelo.galluccio@yahoo.it (A.G.); caparello.giovanna@gmail.com (G.C.); ennioavolio@libero.it (E.A.); emanuelemanes85@gmail.com (E.M.); ferrarosimona@hotmail.it (S.F.); 2 Department of Clinical and Experimental Medicine, University Magna Graecia, 88100 Catanzaro, Italy; 3 School of Specialization in Food Sciences, University of Rome Tor Vergata, 00133 Rome, Italy; 4 Department of Pharmacy, Health and Nutritional Sciences, University of Calabria, 87036 Arcavacata di Rende, Italy; cinzia.giordano@unical.it (C.G.); diego.sisci@unical.it (D.S.); 5 Centro Sanitario, University of Calabria, 87036 Arcavacata di Rende, Italy

**Keywords:** physical activity, Mediterranean diet, lifestyle interventions, eating habits, adolescence

## Abstract

The global pandemic coronavirus disease (COVID-19) resulted in restrictions which forced adolescents to stay at home and influenced their food habits and lifestyles with potential negative health impact. This study aims to investigate the self-reported physical activity (PA) and eating habits related to the consumption of Mediterranean foods in a sample of adolescents during the COVID-19 lockdown enrolled into the DIMENU study. A web survey was launched for 91 adolescents (aged 15–17 years) to assess their adherence to the Mediterranean Diet using the KIDMED test and lifestyle habits using a questionnaire designed following recommendations by Italian National Institute of Health (ISS score). Our results indicate that most of the sample declared no changes in eating habits and PA without sex differences. After dividing the sample into active and sedentary groups based on the self-perceived PA, we found that KIDMED and ISS scores were significantly higher (*p* = 0.0028 and *p* = 0.0001, respectively) in active adolescents. Moreover, KIDMED was positively correlated with ISS only in active adolescents (r = 0.311, *p* = 0.0185). In conclusion, our data underline the impact of the PA on the Mediterranean diet adherence in adolescents during the lockdown, suggesting the usefulness of promoting wellness programs directed towards inactive individuals to increase their awareness on the importance of healthy lifestyles.

## 1. Introduction

On 30 January 2020, the World Health Organization (WHO) Emergency Committee declared a global health emergency based on growing case notification rates at Chinese and international locations caused by a novel coronavirus (COVID-19) [1]. After a few months, COVID-19 had been labeled a global pandemic by the WHO with a decree that provided for the adoption of preventive measures be taken to mitigate the viral spread [2]. In Italy, the procedures for containing the extension of COVID-19 have therefore been implemented with strong implications in the health, social, and economic fields. The public health measures imposed on citizens by the Italian Government include the obligation to practice a social distance (at least 2 m between individuals) and avoid social gatherings, limit contact with elderly individuals and people with poor health, avoid gestures such as handshakes, crowded places, and nonessential meetings [3]. Among these, there was the abrupt interruption of school programs for children and adolescents who by mandate had to remain in their homes during the lockdown aimed at containing and mitigating the spread of COVID-19. Thus, began the so-called “distance learning” in which teachers and students carried out digital teaching and distance learning from their own homes. The same closure also affected other institutional and commercial activities, such as gyms and various fitness and recreational centers. The consequences of these confinement measures led to increased sedentary behaviors, exposing the young and adult population to a greater risk of developing or worsening chronic health conditions [4,5,6]. For adolescents, physical activity (PA) is closely coupled to school-related activities [7]. Therefore, the closure of schools during the COVID-19 pandemic, which also compromises PA participation, caused the risk of longer-term sedentary behaviors. Emerging evidence suggests that PA levels during the pandemic declined in youth [8]. Such necessary restrictions, potentially compromising the lifestyle in terms of physical fitness, also contribute to impacts on eating habits and on maintaining a healthy and varied diet. Indeed, recent studies showed a higher prevalence of inactivity among adolescents during the lockdown, along with changes in number of meals/day and nutritional choices associated with a higher caloric intake [9,10,11]. This situation was particularly serious for maintaining a good state of health in adolescence, because the performance of an optimal level of physical exercise brings many benefits on the parameters of body composition, on the blood profile as well as lipid and carbohydrate metabolism [12]. Since diet is one of the main factors contributing to healthy status [13], it is conceivable that a situation in which the limited availability of food and of daily grocery shopping supply may lead to reduce the consumption of fresh foods in favor of highly processed ones rich in fats, sugars, and salt. Adolescents are in a vulnerable age group requiring careful consideration by caregivers to allow for maintaining health status during the lockdown. In the context of the global COVID-19 pandemic, is important to evaluate the effect of confinement on PA and dietary behaviors covering whole populations or age groups such as in adolescents. Investigating how PA and eating behaviors can be affected by lengthy restrictions is necessary to determine and develop appropriate recommendations in terms of lifestyle modifications for future lockdown policies. Therefore, the aim of this web survey was to examine self-reported physical activity levels and perceived active or sedentary behavior along with the assessment of eating habits related to the consumption of Mediterranean foods in a group of adolescents during the COVID-19 pandemic.

## 2. Materials and Methods

### 2.1. Survey Development

The present online research was developed within the trial DIMENU (Dieta Mediterranea e Nuoto-Mediterranean Diet and Swimming) funded by the EU Regional Operational Program Calabria, Italy (POR Calabria FESR-FSE 2014–2020) (prot.#52243/2017) [12], in which a group of adolescents, aged 15 to 17 years, was recruited from students of the public high school “Istituto Istruzione Superiore”-Castrolibero (CS) and several swim, soccer, and squash teams located in Calabria Region, Italy before the COVID-19 pandemic period. This pilot study was carried out from March to May 2020 in 91 adolescents (42 females and 49 males) who received an invitation by e-mail explaining the purpose of the investigation and inviting recipients to click on the hypertext link to invoke the web browser, presenting the questionnaire. In this survey, 100% of the replies were received. This study was conducted according to the guidelines laid down in the Declaration of Helsinki and approved by the ethics committee of the University of Calabria, Italy (#5727/2018). Written informed consent was obtained from all participants’ parents.

### 2.2. Questionnaire

The questionnaire (DIMENU—Lifestyle Survey, DIMENU-LS) (Table A1 in Appendix A), including 30 questions, was designed to assess changes in both lifestyle and eating habit behaviors during the COVID-19 outbreak. The online questionnaire was divided into three different parts: (1) KIDMED test, used to assess the adherence to the Mediterranean diet (MD) [14], that was updated according to the new MD pyramid by the International Foundation of Mediterranean diet (IFMed) [15]. The MD adherence score, from 0 to 12, was based on a 16-point test. Questions denoting a positive aspect with respect to the MD were assigned a value of +1 (consumption of fruits, vegetables, fish, legumes, whole cereals or grain, nuts, oil, dairy products, and yogurt) and those with a negative connotation −1 (skipping breakfast, consumption of baked goods, sweets, and consumption of delivery junk foods). Level of adherence to MD was indicated as follows: high adherence to MD (≥8 points), medium adherence to MD (4–7 points), low adherence to MD (≤3 points). (2) ISS Score designed following recommendations from “Physical activity guidelines for children and young people” promoted by EpiCentro website belonging to the National Institute of Health (ISS) [16] to counter the increase of sedentary lifestyles and promoting an active lifestyle at home during the period of social isolation. It was used to assess active/sedentary lifestyle of the participants during the COVID-19 lockdown. The ISS score was based on a 7-point test, where from 0 to 3 points indicated a low score and from 4 to 7 points indicated a high score. (3) General questions on the actual perception of changes in lifestyle and eating habits at home. Once completed, each questionnaire was transmitted to the Google platform and the final database was downloaded as a Microsoft Excel sheet.

### 2.3. Statistical Analysis

Data were analyzed by SigmaPlot for Windows Version 12.0 (Systat, San Jose, CA, USA) and reported as mean, standard deviation (SD) and range. Data normality was verified by the Kolmogorov–Smirnov test (with Lilliefors’ correction). The statistical differences between quantitative variables were evaluated by Student’s *t*-test. Qualitative variables were described as frequencies (%) and the statistical differences were evaluated by chi-squared test. The radar charts were built to display the percentage of participants currently adherent to each dietary/lifestyle recommendation respect to the ideal situation (100% compliance). Relationship between variables was evaluated by multivariate linear regression and linear regression analyses. The association between variables is graphically represented through a straight line calculated using the regression coefficient. Pearson’s correlation test was used to assess statistical significance. Statistical significance was set at *p* < 0.05.

## 3. Results

### 3.1. Description of Study Sample

A total of 91 adolescents (aged 15–17 years, 42 females and 49 males), enrolled into the DIMENU study [12], completed the online questionnaire on physical activity behaviors, eating habits changes, eating frequency and adherence to the MD during the lockdown period. A detailed breakdown of the characteristics of the total sample population, divided into girls and boys, is given in Table 1.

Notably, a substantial proportion of participants currently considered their lifestyle active (63%) with the highest ISS score in most of the total sample, which was independent of gender (high score: total sample: 78%, females: 74%, males: 82%). With regards to eating habits during the COVID-19 lockdown, most of the population declared no changes (total sample: 63%, females: 59%, males: 65%), while the percentages of total adolescents who consumed 4 and 5 meals/day were 39% and 26%, respectively. The KIDMED questionnaire, assessing the compliance to the MD recommendations, revealed an average adherence score in total adolescents, again without gender differences, with approximately half of the total population declaring medium adherence to the MD (medium adherence: total sample: 49%, female 48%, male 51%).

### 3.2. Multiple Regression Analysis with KIDMED or Lifestyle and ISS, Age, and Sex

Next, in multiple linear regression we analyzed the KIDMED score as well as lifestyle habits in relation to ISS score using age and sex as independent variables in the total sample. Interestingly, we observed that the KIDMED score can be predicted from a linear combination of the ISS score (β = 0.375, *p* < 0.001), age (β = 0.140 *p* = 0.170), and sex (β = 0.076 *p* = 0.454) (Table 2). Similarly, lifestyle can be predicted from a linear combination of the ISS score (β = 0.432, *p* < 0.001), age (β = −0.087 *p* = 0.373), and sex (β = 0.0463 *p* = 0.642) (Table 2). These data suggest a direct relationship between ISS and both KIDMED and lifestyle, independent of age and sex.

### 3.3. Dietary and Physical Activity Behaviors in Sedentary and Active Adolescents during the Lockdown Period

Based on the self-reported PA behaviors during COVID-19-related restrictions, we divided our population in active and sedentary and compared the percentages of eating habit change, frequency of meals a day, and KIDMED and ISS scores between the two groups of adolescents (Table 3).

**Figure 1:**
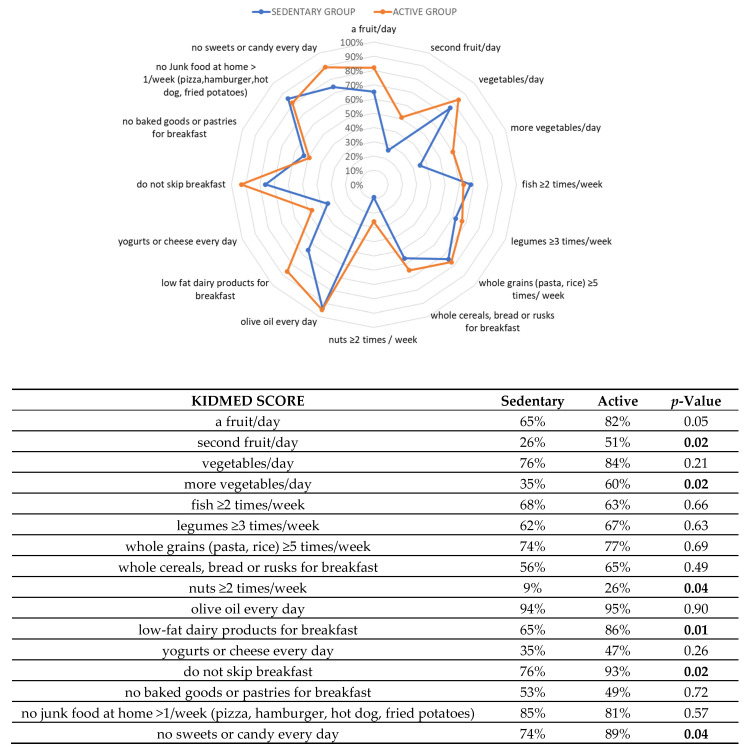
Compliance with items from the KIDMED test in active and sedentary adolescents. The radar chart plots the values of each item of the Mediterranean diet score along a separate axis that starts in the center of the chart (0% compliance) and ends at the outer ring (100% compliance). In the table the statistical differences of each item of the Mediterranean diet score between active and sedentary adolescents were evaluated by chi-squared tests.

As expected, ISS score was significantly higher in active than sedentary adolescents (*p* = 0.0001). Interestingly, in active adolescents a significant higher KIDMED score (*p* = 0.0028) along with a strong increase in the percentage of optimal adherents (*p* = 0.01) and a reduction in poor adherents (*p* = 0.05) to the MD were observed. Moreover, using the results of the KIDMED test, a comparison of the compliance rates for each food was calculated between the two groups and depicted in radar charts which clearly illustrate the gap between the current state (percentage of participants currently adhering to each dietary recommendation) and the ideal situation (100% compliance) (Figure 1). 

**Figure 2:**
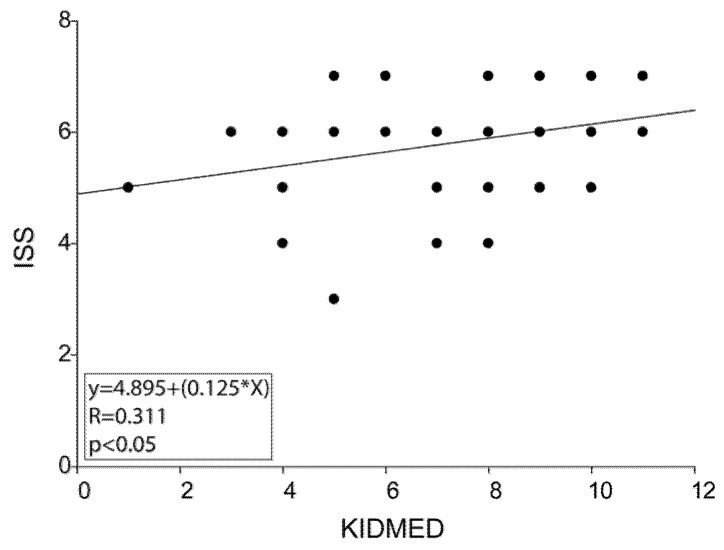
Correlation between ISS score and current lifestyle in active adolescents was analyzed by Pearson’s correlation test. For linear regression graph, the linear equation (y), the correlation coefficient (r), and the statistical significance (*p*) are reported.

There were significant differences between active and sedentary adolescents in the food rates for most of the dietary recommendations. Indeed, active adolescents showed a greater intake of a fruit/day, a second fruit/day, more vegetables/day, nuts ≥ 2 times/week, and low-fat dairy products for breakfast than sedentary. In the same group of adolescents, the percentages of subjects having breakfast and not consuming sweets or candy every day were significantly higher compared to those of other groups (Figure 1). More interestingly, while we found that ISS and KIDMED scores failed to be associated in sedentary adolescents, they were positively correlated in active adolescents (r = 0.311, *p* = 0.0185) (Figure 2).

### 3.4. Self-Perceived Physical Activity and the Relation to Life Satisfaction in Sedentary and Active Adolescents during the COVID-19 Lockdown

We also evaluated the differences in the items of ISS questionnaire in the two PA groups. As shown in Table 4, a strong increase in active compared to sedentary participants was evident in performing PA at least 1 h a day at home (89% vs. 41%, *p* = 0.00001) and at least 1 h 3 times a week (93% vs. 59%, *p* = 0.00007), as well as resting at least 6–8 h in the night (95% vs. 79%, *p* = 0.02) and helping in doing some housework (96% vs. 85%, *p* = 0.05).

Analyzing the PA behaviors into the two groups of active and sedentary adolescents, we asked: “Did you practice physical activity regularly before the COVID-19 emergency or did you gradually start exercising at home?” Table 5 displays results pertaining to reported changes in the PA showing that active adolescents declared to continue physical activity at home in a percentage significantly higher with respect to sedentary (75% vs. 35%, *p* = 0.0001). Similarly, sedentary adolescents declared to not practice exercise at home in a greater percentage than in active group (41% vs. 2%, *p* = 0.00001).

Finally, significant differences between sedentary and active adolescents with respect of their life satisfaction level related to PA were found (Table 6). Indeed, only a minority of active participants considered it boring to practice PA at home compared to sedentary (16% vs. 44%, *p* = 0.003) and most of them declared to feel good (active: 58% vs. sedentary: 32%, *p* = 0.01).

## 4. Discussion

In the present web survey, we investigated the self-perceived lifestyle, including PA intensity levels, and eating habits among a cohort of adolescents during the lockdown period caused by the COVID-19 pandemic. Globally, most of the participants (63% of total population) declared an active current lifestyle and no changes with regards of eating habits without gender differences. Interestingly, using the KIDMED questionnaire to assess both the score to the MD and the compliance to the MD recommendations, we observed in all adolescents, as well as in females and in males, an average adherence to the MD. This result confirms our previous investigations on MD adherence conducted in a pre-lockdown period on the same group of adolescents and on a sample of adults living in the same Mediterranean area [12,17]. Similarly, Dragun et al. reported no substantial differences in dietary habits between pre-lockdown and lockdown period, including the overall adherence to the MD and their food choices [18]. In other observations, conflicting data are reported because COVID-19 confinement influenced dietary habits, particularly in younger populations [19]. In fact, data collected by an anonymous online questionnaire on food intake among a group of adolescents from Spain, Italy, Brazil, Colombia, and Chile showed a modified consumption in certain types of foods, such as fried food, sweet food, legumes, vegetables, and fruits which were significantly increased during the lockdown [11]. Moreover, the school closures, coupled with additional socio-behavioral adaptations (e.g., social distancing, quarantining), had a strong impact on the lifestyle activities of children and adolescents across the whole day leading to significant decreases in PA along with increases in sedentary behavior, and disrupted sleep quality in children and adolescents [20]. As is well known, PA is associated with numerous health benefits for adolescents, including cardio-metabolic effects, motor skill development, bone density, and emotional regulation/psychological health [21,22]. Specifically, guidelines and recommendations provided by the WHO for children and adolescents reported that they should participate in moderate-to-vigorous PA at least 1 h per day mostly aerobic, physical activity, across the week, or they should practice vigorous-intensity aerobic activities at least 3 days a week [23]. Instead, sedentary behaviors indicated often as “screen-based media use behaviors”, including watching television (TV), using computers/smartphones, and playing video games are associated with various negative health consequences [24,25]. The adverse consequences resulting from sedentary behaviors include an increased risk of obesity, cardiovascular disease, and all-cause mortality [26]. For that reason, based on guidelines issued by the WHO, the Italian National Institute of Health adapted the recommended levels of PA for different age groups, including in adolescents aged 12–17 years during the COVID-19 outbreak emergency in our country [16,27]. During lockdown, helping to increase the sense of self-efficacy and improve general health of our population, we published a short leaflet and a number of specific suggestions on how to eat well and exercise at home [28]. We have also created an official website of DIMENU project [29] and a Facebook page [30] as innovative ways for assuring additional support and information on health-related issues. Notably, in our study, most adolescents, who declared that their lifestyle was active during the lockdown, have also the highest level of ISS score confirming the self-perceived PA levels. Similarly, as also reported by another recent survey on students during social confinement, it was observed that adolescents tried to carry out PA, getting interested in cooking, reading, and playing board games at home as well [31]. After dividing the population into the active and sedentary groups, we found that active adolescents closely adhered to food- and nutrient-based recommendations. Particularly, we found significant differences in the consumption of some typical Mediterranean foods, such as “a fruit/day”, “more vegetables”, “a second fruit portion/day”, “nuts weekly”, “low-fat dairy products for breakfast” as well as in “skipping breakfast” and “no sweets or candy every day”. Conflicting data are reported on the association between skipping breakfast and overweight/obesity in adolescents [32]. Thus, further studies are needed to clarify the effects of breakfast on weight management. Our results are in line with other studies in which an increase of consumption of junk food, sweets, and candy were related to the sedentary behavior [9]. In addition, based on our data we highlight the impact of PA on the adherence to the MD and on a subjective well-being and healthy lifestyle in adolescents. In this context, we have recently described that PA associated with a nutrition education program improves the adherence to the MD and reduces systemic inflammation in adolescents, suggesting that their combined promotion represent a strategy for assuring the prevention and control of chronic non-communicable diseases over the entire lifespan [33]. Experiences from previous epidemics have also shown that there is a need to maintain optimal nutrition at individual and global levels, in order to positively influence the physical and mental health of the population [34]. In this sense, the knowledge of the dietary and lifestyle habits in each age range is necessary to promote MD patterns and regular PA as a priority public health action after COVID-19 confinement or to develop future reactions to unavoidable pandemics.

This study includes some limitations, such as the small sample size and the online survey. Although a large study population may strengthen the results, in our web survey conducted over a short timeframe a small group of subjects interviewed was sufficient to quickly obtain a picture of adolescent behavior. Regarding the online questionnaire that we used to perform this study, this method does not permit achieving a comparable response quality respect to traditional ones, however, during the lockdown period it represented a unique alternative approach for data collection. We also used a website to promote MD pattern and foster to increase PA intensity levels [29].

## 5. Conclusions

In summary, data from our web survey report that active adolescents have higher adherence to the MD, a subjective well-being and life satisfaction level related to PA respect to sedentary underlining the impact of the PA intensity level on the eating habits and healthy behaviors during the lockdown. These findings suggest the usefulness of planning effective education programs including energy and nutritionally balanced diets as well as regular PA, as a part of wellness programs and of interventions in adolescence for a healthy lifestyle.

## Figures and Tables

**Table 1:** General characteristics of our study population.

Characteristics	Total Sample	Girls	Boys
**Subjects (number)**	91	42 (46%)	49 (54%)
**Age (years ± SD, range)**	16.60 ± 1.28 (13–19)	16.83 ± 1.27 (14–18)	16.40 ± 1.26 (13–19)
**Current lifestyle **			
Sedentary	34 (37%)	18 (20%)	16 (17%)
Active	57 (63%)	25 (26%)	32 (37%)
**ISS score (M ± SD, range)**	5.37 ± 1.19 (1–7)	5.23 ± 1.1 (1–7)	5.49 ± 1.27 (3–7)
Low (0–3)	20 (22%)	11 (26%)	9 (18%)
High (4–7)	71 (78%)	31 (74%)	40 (82%)
**Eating habits change **	34 (37%)	17 (41%)	17 (35%)
**Meals/day **			
1–2 meals/day	4 (4%)	0	4 (8%)
3 meals/day	28 (31%)	15 (36%)	13 (27%)
4 meals/day	35 (39%)	15 (36%)	20 (41%)
5 meals/day	24 (26%)	12 (28%)	12 (24%)
**KIDMED score (M ± SD, range)**	6.52 ± 2.45 (0–11)	6.28 ± 2.68 (1–11)	6.73 ± 2.24 (0–11)
Optimal adherence (≥8)	37 (41%)	16 (38%)	21 (43%)
Medium adherence (4–7)	45 (49%)	20 (48%)	25 (51%)
Poor adherence (≤3)	9 (10%)	6 (14%)	3 (6%)

**Table 2:** Multiple linear regression analysis between KIDMED as well as lifestyle habits and a set of different variables.

*Variables*		KIDMED		Lifestyle Habits		
	β	*se*	*p*-Value	β	*se*	*p*-Value
**ISS**	0.375	0.206	**<0.001**	0.432	0.039	**<0.001**
**Age**	0.140	0.194	0.170	−0.087	0.037	0.373
**Sex**	0.076	0.491	0.454	0.045	0.093	0.642
	R = 0.388	R^2^ = 0.151	Adj R^2^ = 0.122	R = 0.463	R^2^ = 0.215	Adj R^2^ = 0.188

For each variable, the linear regression coefficient (β), the standard error (*se*), adjusted R-squared (Adj R^2^), and the statistical significance (*p*-value) are reported.

**Table 3:** Dietary and physical activity habits in sedentary and active adolescents during the lockdown.

Characteristics	Total Sample (*n* = 91)		*p*-Value
	Sedentary (*n* = 34)	Active (*n* = 57)	
**Age (years ± SD, range)**	16.88 ± 1.26 (15–19)	16.43 ± 1.28 (13–19)	0.1063
**ISS score (M ± SD, range)**	4.67 ± 1.22 (1–7)	5.78 ± 1.2 (3–7)	**0.0001**
Low (0–3)	6 (18%)	1 (2%)	**0.005**
High (4–7)	28 (82%)	56 (98%)	**0.005**
**Eating habits change **	16 (47%)	18 (31%)	0.1398
**Meals/day **			
1–2 meals/day	2 (6%)	2 (4%)	0.5931
3 meals/day	12 (35%)	16 (28%)	0.4701
4 meals/day	13 (38%)	22 (39%)	0.9727
5 meals/day	7 (21%)	17 (30%)	0.3334
**KIDMED score** **(M ± SD, range)**	5.5 ± 2.47 (0–10)	7.14 ± 2.45 (1–11)	**0.0028**
Optimal adherence (≥8)	8 (23%)	29 (51%)	**0.01**
Medium adherence (4–7)	20 (59%)	25 (44%)	0.1672
Poor adherence (≤3)	6 (18%)	3 (5%)	**0.05**

Statistical differences were evaluated by Student’s *t*-test and by chi-squared tests.

**Table 4:** Percentages (%) of sedentary and active adolescents during lockdown with respect of items from the ISS questionnaire.

ISS Questionnaire	Sedentary	Active	*p*-Value
Are you exercising at least 1 h a day at home?	14 (41%)	51 (89%)	**0.00001**
Currently in your free time, do you spend a maximum of 2 h a day on your PC/tablet/TV/smartphone? (excluding the time, you dedicate to studying)	21 (62%)	44 (77%)	0.1150
Is physical activity currently performed at home (at least 1 h) 3 times a week?	20 (59%)	53 (93%)	**0.00007**
Having to stay at home all day, do you respect a night’s rest of at least 6–8 h?	27 (79%)	54 (95%)	**0.02**
Are you dedicating time to some hobbies? (music, reading, drawing, creative activities)	28 (82%)	50 (88%)	0.4791
Are you helping the family with some housework? (dusting, making the bed, setting/clearing the table)	29 (85%)	55 (96%)	**0.05**
Are you using the web to integrate your study activity with other cultural activities?	18 (53%)	22 (39%)	0.1822

Statistical differences were evaluated by chi-squared tests.

**Table 5:** Reported changes (%) in physical activity behavior during the lockdown by sedentary and active adolescents.

ITEMS	Sedentary	Active	*p*-Value
Yes, I practiced physical activity regularly and I am continuing it at home	12 (35%)	43 (75%)	**0.0001**
Yes, I practiced physical activity regularly but I’m NOT continuing it at home	14 (41%)	1 (2%)	**0.00001**
Yes, I gradually started exercising at home	7 (21%)	12 (21%)	0.9579
None of the above	1 (3%)	1 (2%)	0.8833

Statistical differences were evaluated by chi-squared tests.

**Table 6:** Percentages (%) of sedentary and active adolescents with respect of their consideration of physical activity practiced at home during the lockdown.

In this Period, How Do You Consider Physical Activity Practiced at Home?	Sedentary	Active	*p*-Value
Tiring	3 (9%)	2 (3%)	0.2817
Boring	15 (44%)	9 (16%)	**0.003**
It makes me feel good	11 (32%)	31 (58%)	**0.01**
It temps me to continue in the sport	5 (15%)	13 (23%)	0.3479

Statistical differences were evaluated by chi-squared tests.

## Data Availability

The datasets used and/or analyzed during the current study are available from the corresponding author on reasonable request.

## Appendix A

**Table A1:** DiMeNu—Lifestyle survey during the COVID-19 lockdown (DiMeNu-LS).

	KIDMED SCORE		
**1.**	Takes a fruit or fruit juice every day		Yes/No
**2.**	Has a second fruit every day		Yes/No
**3.**	Has fresh or cooked vegetables regularly once a day		Yes/No
**4.**	Has fresh or cooked vegetables more than once a day		Yes/No
**5.**	Consumes fish regularly (≥2 times/week)		Yes/No
**6.**	Likes legumes and eats them ≥3 times/week		Yes/No
**7.**	Consumes whole grains (pasta or rice) almost every day or ≥5 times/week		Yes/No
**8.**	Has whole cereals or grains (bread or rusks) for breakfast		Yes/No
**9.**	Consumes nuts regularly (every day)		Yes/No
**10.**	Uses olive oil at home		Yes/No
**11.**	Has a low-fat dairy product for breakfast (yogurt, milk, etc.)		Yes/No
**12.**	Takes two yogurts and/or some cheese (40 g) daily		Yes/No
**13.**	Skips breakfast		Yes/No
**14.**	Has commercially baked goods or pastries for breakfast		Yes/No
**15.**	Goes > 1/week to a fast-food restaurant (hamburger)		Yes/No
**16.**	Takes sweets and candy several times every day		Yes/No
	**ISS SCORE**		
**17.**	Are you exercising at least 1 h a day at home?		Yes/No
**18.**	Currently in your free time, do you spend a maximum of 2 h a day on your PC/tablet/TV/smartphone? (excluding the time you dedicate to studying)		Yes/No
**19.**	Is physical activity currently performed at home (at least 1 h) 3 times a week?		Yes/No
**20.**	Having to stay at home all day, do you respect a night’s rest of at least 6–8 h?		Yes/No
**21.**	Are you dedicating time to some hobbies? (music, reading, drawing, creative activities)		Yes/No
**22.**	Are you helping the family with some housework? (dusting, making the bed, setting the table)		Yes/No
**23.**	Are you using the web to integrate your study activity with other cultural activities?		Yes/No
	**PERCEPTION OF CHANGES IN LIFESTYLE AND EATING HABITS AT HOME.**		
**24.**	How do you define your lifestyle currently?	Active Sedentary	
**25.**	In this period, how do you consider physical activity practiced at home?	Tiring Boring It makes me feel good It temps me to continue in the sport	
**26.**	During this period does your eating habits changed?	Yes/No	
**27.**	During this period how many meals/day do you have?	1–2 meal/day 3 meals/day 4 meals/day 5 meals/day	
**28.**	Do you drink at least 2 L/day of water?	Yes/No	
**29.**	Before COVID-19 emergency did you practice regularly physical activity or did you gradually start exercising at home?	Yes, I practiced physical activity regularly and I am continuing it at home Yes I practiced physical activity regularly but I’m NOT continuing it at home Yes I gradually started exercising at homeNone of the above	
**30.**	What kind of physical activity are you doing?	Bodyweight exercises/jumping rope/running or tapis roullant or cyclette/abdominal exercises/step/exercise with objects at home/squat/or lunges or buttocks or legs exercises/push-ups/exergame/stretching/weight training	
